# ICTV Virus Taxonomy Profile: Potyviridae 2022

**DOI:** 10.1099/jgv.0.001738

**Published:** 2022-05-05

**Authors:** Alice K. Inoue-Nagata, Ramon Jordan, Jan Kreuze, Fan Li, Juan José López-Moya, Kristiina Mäkinen, Kazusato Ohshima, Stephen J. Wylie, ICTV Report Consortium

**Affiliations:** 1 Embrapa Vegetables, Brasília, Brazil; 2 USDA, Beltsville, MD, USA; 3 CIP, Lima, Peru; 4 Yunnan Agricultural University, Yunnan, PR China; 5 CRAG-CSIC, Barcelona, Spain; 6 University of Helsinki, Helsinki, Finland; 7 Saga University, Saga, Japan; 8 Murdoch University, Perth, Australia

**Keywords:** ICTV Profile, taxonomy, *Potyviridae*

## Abstract

The family *Potyviridae* includes plant viruses with single-stranded, positive-sense RNA genomes of 8–11 kb and flexuous filamentous particles 650–950 nm long and 11–20 nm wide. Genera in the family are distinguished by the host range, genomic features and phylogeny of the member viruses. Most genomes are monopartite, but those of members of the genus *Bymovirus* are bipartite. Some members cause serious disease epidemics in cultivated plants. This is a summary of the International Committee on Taxonomy of Viruses (ICTV) Report on the family *Potyviridae*, which is available at ictv.global/report/potyviridae.

## Virion

**Fig. 1.**
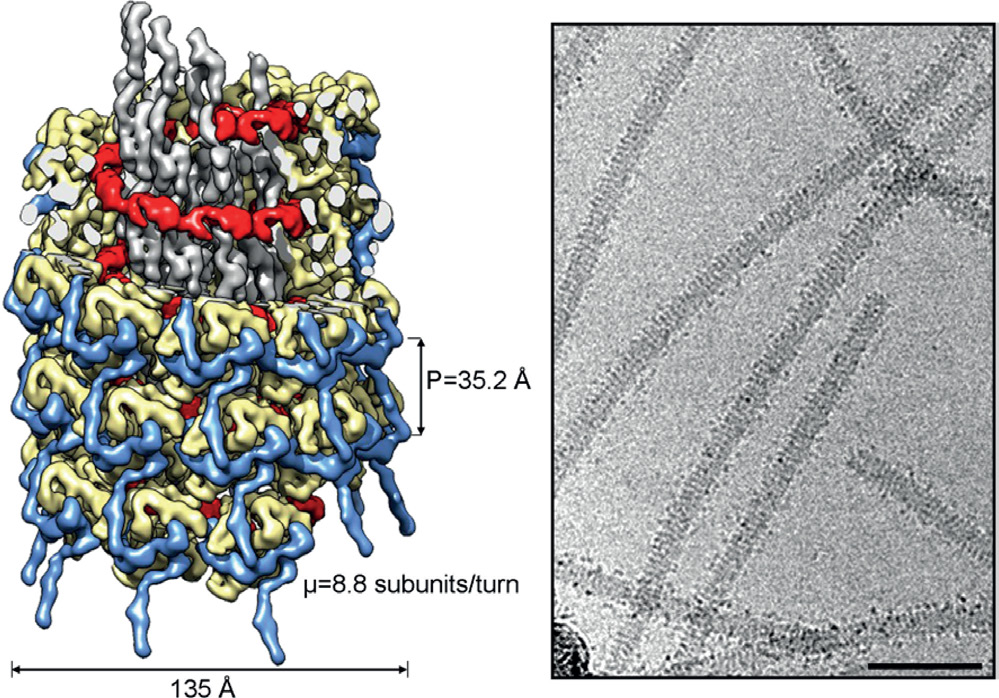
(Left) Cryo-electron microscopy reconstruction of a section of a virion of turnip mosaic virus (TuMV). Depicted are the ssRNA (red), and the central globular domain (pale yellow), N-terminal arm (blue) and C-terminal region (grey) of the capsid protein. In the upper section some of the densities are cut-away. (Right) Cryo-electron micrograph of TuMV virions where the periodic organization of the helical filaments is apparent. Bar, ~500 nm. Images are courtesy of R. Cuesta and M. Valle, CIC bioGUNE, Spain, based on [7].

The flexuous, filamentous particles are typically 650–950 nm long and 11–20 nm wide, with helical symmetry and a pitch of about 3.4–3.5 nm (Fig. 1, Table 1), but members of the genus *Bymovirus* are bipartite with particles of two modal lengths of 250–300 nm and 500–600 nm. Virions contain a core capsid protein (CP) of 28.5–47 kDa [1]; the tip of the particle may contain the virus-encoded proteins genome-linked protein (VPg), helper-component protease (HC-Pro), cylindrical inclusion (CI) and the host factor elF4E. There are serological relationships among many members.

**Table 1.** Characteristics of members of the family *Potyviridae*

Example	potato virus Y-O (U09509), species *Potato virus Y*, genus *Potyvirus*
Virion	Non-enveloped, flexuous and filamentous particles, 650–950 nm long and 11–20 nm in diameter with a core capsid protein
Genome	8.2–11.5 kb of positive-sense, single-stranded, usually monopartite RNA (bipartite in the genus *Bymovirus*)
Replication	Cytoplasmic, initiated in virus replication complexes on 6K2-induced membranous vesicles at endoplasmic reticulum exit sites
Translation	Directly from genomic RNA
Host range	Plants; transmission by arthropods, plasmodiophorids (*Bymovirus*), seeds or pollen
Taxonomy	Realm *Riboviria*, kingdom *Orthornavirae*, phylum *Pisuviricota*, class *Stelpaviricetes*, order *Patatavirales*: >10 genera including >230 species

## Genome

**Fig. 2.**
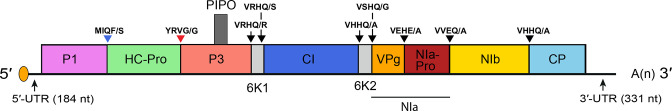
Schematic diagram of the genome organization of potato virus Y. VPg, virus protein genome-linked (indicated by an ellipse at the 5′-terminus); P1, protein 1 protease; HC-Pro, helper component protease; P3, protein 3; PIPO, pretty interesting *Potyviridae* ORF; 6K1, 6 kDa peptide 1; CI, cylindrical inclusion; 6K2, 6 kDa peptide 2; NIa-Pro, nuclear inclusion a-protease; NIb, nuclear inclusion b, RNA-directed RNA polymerase; CP, capsid protein. Arrowheads indicate cleavage sites of P1 (blue), HC-Pro (red) and NIa-Pro (black).

The positive-sense ssRNA genome ranges from 8.2 kb (members of the species *Artichoke latent virus*, genus *Macluravirus*, and species *Bellflower veinal mottle virus*, genus *Bevemovirus*) to 11.5 kb (species *Celery latent virus*, genus *Celavirus*), with a VPg covalently linked to the 5′-end, and a polyadenylated 3′-terminus (Fig. 2). The genome of the celavirus celery latent virus is not polyadenylated. Most genomes are monopartite, but those of members of the genus *Bymovirus* are bipartite. The major large ORF of monopartite genomes encodes a polyprotein that is cleaved into functional proteins at semi-conserved sites by two or three viral proteases [2]. Bipartite *Bymovirus* genomes encode two polyproteins that are cleaved by two proteases. A second small ORF, PIPO, is generated by a polymerase slippage mechanism and is expressed as the *trans*-frame protein P3N-PIPO [3]. Another additional small ORF, PISPO, is generated through a transcriptional slippage mechanism among sweet potato-subgroup potyviruses and leads to the production of a *trans*-frame protein P1N-PISPO. Some viruses lack one or both of the P1 and HC-Pro N-terminal coding regions, and may be replaced by genus-specific or species-specific regions.

## Replication

Genomic RNA serves as a template for both translation and replication (reviewed by [4]). The genomic RNA replicates via the production of a full-length negative-sense RNA, which takes place in the cytoplasmic viral replication complex (VRC). The VRC is formed by virus proteins, such as NIb, 6K2, VPg, NIa-Pro, HC-Pro, CI, and possibly P3 and 6K1, and host factors. The viral genome is transported cell-to-cell through the plasmodesmata, probably in the form of particles, involving the coordinated action of CP, CI, P3, 6K2 and P3N-PIPO [5].

## Taxonomy

Current taxonomy: ictv.global/taxonomy. Members of different genera in the family are distinguished by host range, vector, genomic features and phylogeny, with species demarcation typically based upon sequence identity of the large ORF (or, if necessary, the CP-coding region) being <76 % (nt) and <82 % (aa) [6]. Thresholds for other coding regions are usually 58 % (P1 coding region) or 74–78 % (other regions). Members of the family *Potyviridae* are transmitted by aphids, eriophyid mites, whiteflies and plasmodiophorids, but the vector is not known for members of five genera. New species or genera in the family will be considered after analyses based on complete genome sequences and additional data about biological characteristics.

## Resources

Full ICTV Report on the family *Potyviridae*: ictv.global/report/potyviridae.

## Abbreviations

CI: cylindrical inclusion
CP: capsid protein
HC-Pro: helper-component protease
VPg: virus protein genome-linked
VRC: viral replication complex
